# Tris(1,3-dichlorpropyl)phosphat – Bestimmung von Bis(1,3-dichlorpropyl)phosphat in Urin mittels LC-APCI-ESI-MS/MS: Biomonitoring-Methode

**DOI:** 10.34865/bi72236d9_4or

**Published:** 2024-12-23

**Authors:** Petra Krystek, Henry Beeltje, Marc Machiel George Houtzager, Eric Martin van den Hoeven, Laura Kuhlmann, Elisabeth Eckert, Thomas Göen, Andrea Hartwig, MAK Commission

**Affiliations:** 1 TNO – Location Utrecht. Environmental Monitoring. Sensing and Analysis P.O. Box 80015 NL-3508 TA Utrecht Niederlande; 2 Friedrich-Alexander-Universität Erlangen-Nürnberg. Institut und Poliklinik für Arbeits-, Sozial- und Umweltmedizin Henkestraße 9–11 91054 Erlangen Deutschland; 3 Institut für Angewandte Biowissenschaften. Abteilung Lebensmittelchemie und Toxikologie. Karlsruher Institut für Technologie (KIT) Adenauerring 20a, Geb. 50.41 76131 Karlsruhe Deutschland; 4 Ständige Senatskommission zur Prüfung gesundheitsschädlicher Arbeitsstoffe. Deutsche Forschungsgemeinschaft, Kennedyallee 40, 53175 Bonn, Deutschland. Weitere Informationen: Ständige Senatskommission zur Prüfung gesundheitsschädlicher Arbeitsstoffe | DFG

**Keywords:** TDCPP, BDCPP, Flammschutzmittel, Biomonitoring, Urin, LC-APCI-ESI-MS/MS

## Abstract

The working group “Analyses in Biological Materials” of the German Senate Commission for the Investigation of Health Hazards of Chemical Compounds in the Work Area (MAK Commission) developed and verified this biomonitoring method for the determination of urinary concentrations of bis(1,3-dichloropropyl) phosphate (BDCPP), which is the major metabolite of tris(1,3-dichloropropyl) phosphate (TDCPP). TDCPP is one of the most commonly used organophosphate flame retardants in cars, residential furniture, and products containing polyurethane foam, and has been detected in dust from private houses, office buildings, and car interiors, suggesting that a majority of the general population is exposed to TDCPP. The aim of this work was to establish a reliable, selective, and sensitive method for the detection of BDCPP in urine. The urine samples are spiked with the internal standard d_10_-BDCPP and slightly acidified. Cleanup by mixed-mode anion-exchange solid-phase extraction is applied, and BDCPP is detected by liquid chromatography with simultaneous atmospheric pressure chemical ionisation and electrospray ionisation-tandem mass spectrometry. Calibration is carried out in ultra-pure water ∶ MeOH (4 ∶ 1, v/v). Good precision data with standard deviations below 7%, as well as good accuracy data with mean relative recoveries in the range of 93.6–101%, show that the method provides reliable and accurate analytical results. The method is both selective and sensitive, and the limit of quantitation of 0.2 ng BDCPP/l urine is sufficient to determine occupational exposure as well as higher background exposure levels to TDCPP in the general population.

## 1 Kenndaten der Methode

**Table:** 

**Matrix**		Urin	
**Analytisches Messprinzip**		Flüssigkeitschromatographie mit simultaner chemischer Ionisation bei Atmosphärendruck und Elektrospray-Ionisation-Tandem-Massenspektrometrie (LC‑APCI‑ESI‑MS/MS)	
**Parameter und entsprechender Arbeitsstoff**			
**Arbeitsstoff**	**CAS‑Nr.**	**Parameter**	**CAS‑Nr.**
Tris(1,3‑dichlorpropyl)phosphat (TDCPP)	13674-87-8	Bis(1,3‑dichlorpropyl)phosphat (BDCPP)	72236-72-7

### Zuverlässigkeitskriterien

#### BDCPP

**Table:** 

Präzision in der Serie:	Standardabweichung (rel.)	*s_w_* = 6,5 %, 3,1 % bzw. 5,5 %
	Streubereich	*u* = 15,4 %, 7,3 % bzw. 13,0 %
	bei einer dotierten Konzentration von 0,9 μg, 9 μg oder 90 μg BDCPP pro Liter Urin und n = 8 Bestimmungen	
Präzision von Tag zu Tag:	Standardabweichung (rel.)	*s_w_* = 8,5 %, 8,7 % bzw. 5,5 %
	Streubereich	*u* = 20,1 %, 20,6 % bzw. 13,0 %
	bei einer dotierten Konzentration von 0,9 μg, 9 μg oder 90 μg BDCPP pro Liter Urin und n = 8 Bestimmungen	
Richtigkeit:	Wiederfindung (rel.)	*r* = 93,6 %, 99,5 % bzw. 100,5 %
	bei einer dotierten Konzentration von 0,9 μg, 9 μg oder 90 μg BDCPP pro Liter Urin und n = 8 Bestimmungen	
Nachweisgrenze:	0,06 μg BDCPP pro Liter Urin	
Bestimmungsgrenze:	0,2 μg BDCPP pro Liter Urin	

## 2 Allgemeine Informationen zu TDCPP

Tris(1,3‑dichlorpropyl)phosphat (TDCPP) ist eines der am häufigsten in Weichschaumstoffen für die Automobilindustrie verwen­deten Organophosphat-Flammschutzmittel (*organophosphate flame retardants*, OPFRs). Es ist bei Raum­temperatur flüssig und weist einen niedrigen Dampfdruck auf (5,6 × 10^–6^ Pa bei 25 °C) (OECD 2009). Der typische Massenanteil an TDCPP in z. B. Polyurethanschaum liegt im Bereich von 5–10 Gew.‑%. Eine geringere, aber immer noch bedeutende Menge TDCPP wird auch in Weichschaumstoffen in Möbeln für den privaten Wohnbereich eingesetzt (van der Veen und de Boer 2012). Da es sich bei TDCPP um ein additives Flammschutzmittel handelt, kann es bis zu einem gewissen Grad aus dem behandelten Material herausdiffundieren.

In neueren Studien wurde TDCPP im Staub in Wohngebäuden, Bürogebäuden und Kraftfahrzeugen nachgewiesen, was darauf hindeutet, dass ein Großteil der Allgemeinbevölkerung chronisch gegen TDCPP exponiert ist. Zu einer beruflichen Exposition kann es bei der Produktion von TDCPP, bei der Herstellung und Verarbeitung von Weichschaumstoff und Verbundschaum sowie bei der Herstellung von Autobauteilen kommen (EU 2008). Zudem können auch Feuerwehrleute gegen TDCPP exponiert sein (Jayatilaka et al. 2017).

Bei einer TDCPP-Exposition können verschiedene Expositionswege von Bedeutung sein (Carignan et al. 2013; Norén et al. 2017). In einer unveröffentlichten In‑vitro‑Studie zur dermalen Resorption wurde Haut vom Menschen acht Stunden lang gegen ^14^C‑TDCPP exponiert, um die Aufnahme während eines Arbeitstages nachzustellen. Die mittlere dermale Gesamtresorption betrug 15,4 %, 10,69 % und 6,0 % bei einer applizierten Dosis von 0,003 mg, 0,01 mg bzw. 0,12 mg TDCPP/cm² Haut (EU 2008). Nach oraler und inhalativer Exposition kann von einer 100%igen Resorption ausgegangen werden (EU 2008). Kinetik- und Verteilungsstudien an Ratten haben ergeben, dass aufgenommenes TDCPP schnell verstoffwechselt und ausgeschieden wird und keine nennenswerte Akkumulation im Körper zu erwarten ist (Krystek et al. 2019).

Nomeir et al. (1981) zeigten in einer Studie mit Ratten, dass der Aufnahmeweg von radioaktiv markiertem TDCPP kaum Auswirkungen auf dessen Verteilung im Organismus hatte. Die gastrointestinale Resorption und Verteilung blieb zudem über einen Dosisbereich von zwei Größenordnungen unverändert. ^14^C‑TDCPP wurde schnell metabolisiert und innerhalb der ersten 24 Stunden waren mehr als 80 % der Radioaktivität mit dem Urin oder den Faeces ausgeschieden oder als ^14^CO_2_ abgeatmet. Der quantitativ bedeutsamste Metabolit im Urin war Bis(1,3‑dichlorpropyl)­phosphat (BDCPP) (Nomeir et al. 1981). Zur gleichen Zeit und ebenfalls an Ratten untersuchten auch Lynn et al. (1981) die Verteilung, Verstoffwechselung und Ausscheidung von TDCCP. Fünf Tage nach intravenöser Verabreichung von ^14^C‑TDCPP waren 92 % der verabreichten Dosis mit dem Urin (54 %), den Faeces (16 %) und der Ausatemluft (22 %, als ^14^CO_2_) ausgeschieden, während 4 % im Körper wiedergefunden wurden. BDCPP wurde im Urin, in den Faeces und in der Galle als Hauptmetabolit identifiziert (Lynn et al. 1981). Auch in einer weiteren Studie, in der der Metabolismus von TDCPP in vitro mit Leberfraktionen vom Menschen sowie in vivo an Ratten untersucht wurde, war BDCPP der Hauptmetabolit (Van den Eede et al. 2013 a).

**Abb.1:**
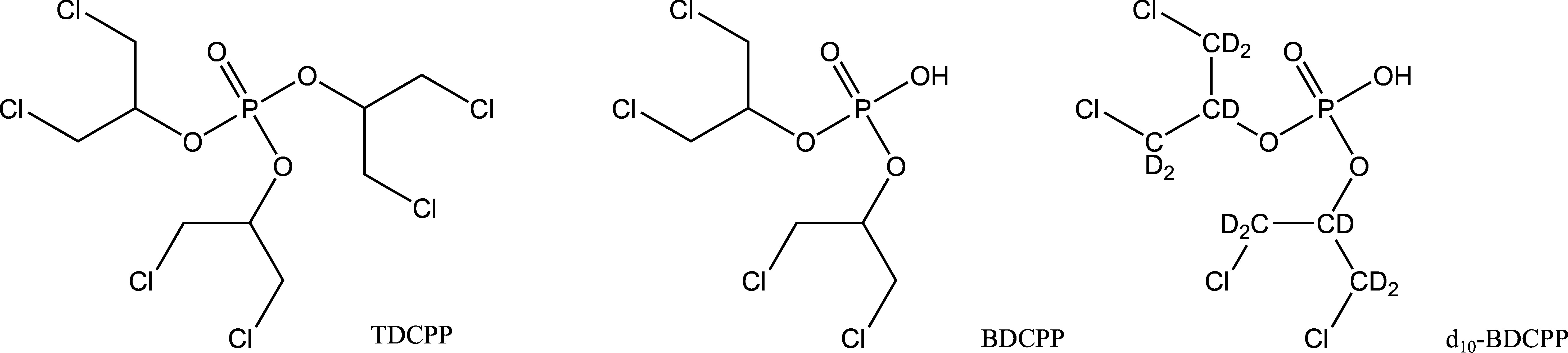
Strukturformeln von TDCPP, BDCPP und d_10_‑BDCPP (interner Standard)

Da kein anderes bislang bekanntes OPFR zu BDCPP verstoffwechselt wird (EU 2008), ist BDCPP der am besten geeig­nete Metabolit für das Human-Biomonitoring von TDCPP (Abbildung 1). Nach der harmonisierten Einstufung und Kennzeichnung steht dieser Stoff im Verdacht, Krebs zu verursachen (Europäische Kommission 2018). Eine Bewertung von TDCPP durch die Kommission ist bislang nicht erfolgt. In der Literatur publizierte BDCPP-Konzentrationen im Urin der Allgemeinbevölkerung sowie im Urin von potenziell expo­nierten Arbeitern sind in Tabelle 1 und Tabelle 2 aufgeführt.

**Tab.1:** BDCPP im Urin der beruflich nicht belasteten Allgemeinbevölkerung

Studienkollektiv (Land; Alter; n)	NWG [μg/l]	BG [μg/l]	BDCPP [μg/l]		Literatur
			Mittelwert ± Standardabweichung	Bereich	
Erwachsene (Belgien; Alter k. A.; 14)	0,04	0,15	0,29 ± 0,27 0,19 (Median)	0,06–0,90 (DF = 100)	Bastiaensen et al. 2018
Erwachsene (Belgien; 20–50 a; 10)	–	0,05	0,11 (Median)^a)^; 0,19 (Median)^b)^	0,03–4,58^a)^ (DF =66,3)^a)^; 0,06–10,79^b)^ (DF = 100)^b)^	Bastiaensen et al. 2021
Erwachsene Frauen (USA; 18–46 a; 31^c)^)	0,056	–	0,83 (GM)^d)^	0,06–9,87 (DF = 100)	Carignan et al. 2017
Erwachsene Frauen (USA; 18–46 a; 30^e)^)			0,69 (GM)^d)^	< NWG–10,38 (DF = 97)	
Erwachsene (Taiwan; > 17 a; 317)	–	0,02	0,69	0,48–0,89^f)^ (DF = 90)	Cheng et al. 2023
Erwachsene (USA; 23–46 a; 9)	0,008	–	0,148 (GM) 0,083 (Median)	0,046–1,662 (DF = 100)	Cooper et al. 2011
Erwachsene (USA; Alter k. A.; 16)	0,02	–	0,46 0,09 (Median)	< NWG–3,9 (DF = 94)	Dodson et al. 2014
Erwachsene (USA; 19–67 a; 53)	–	–	0,37 (GM)^d)^	< NWG–4,46 (DF = 83,0)	Hoffman et al. 2015
Erwachsene Männer mit Fruchtbarkeitsproblemen (USA; 18–54 a; 355 Proben von 220 Männern)	0,00002–0,00011	–	0,62 (GM)^d)^	< NWG–10,3 (DF = 85,1)	Ingle et al. 2018
Erwachsene (USA; Alter k. A.; 76)	0,11	–	0,69 (Median)	0,31–6,8 (DF = 100)	Jayatilaka et al. 2017
Erwachsene (Niederlande; Alter k. A.; 40)	0,06	0,2	0,14	< NWG–0,66 (DF = 33,3)	Krystek et al. 2019
Erwachsene (China; 20–80 a; 1863)	0,06	–	0,10 (Median)^d)^	< LOD–0.23 (DF = 60,7)	Liu et al. 2024
Erwachsene Männer (USA; 28–46 a; 33); Urinsammlung am Nachmittag	0,0327	–	0,384 (GM)	(DF = 100)	Meeker et al. 2013 a
Erwachsene Männer (USA; 28–46 a; 33); Urinsammlung am Vormittag			0,122 (GM)		
Erwachsene Männer (USA; 18–54 a; 45)	0,0327	–	0,13 (GM) 0,12 (Median)	< NWG–25,0 (DF = 91)	Meeker et al. 2013 b
Erwachsene (Inuit aus Kanada; > 16 a; 28; Probenahme in 2017)	–	0,05	0,53 0,49 (GM) 0,45 (Median)	0,27–1,70 (DF = 100)	Nero et al. 2024
Erwachsene (Inuit aus Kanada; > 16 a; 1367; Probenahme in 2018–2019)			1,1 0,46 (GM) 0,49 (Median)	< NWG–4,00 (DF = 94,4)	
Erwachsene (USA; Alter k. A.; 13)	0,025	0,1	3,4 ± 2,5 2,5 (GM) 2,4 (Median)	0,5–7,3 (DF = 100)	Petropoulou et al. 2016
Erwachsene Frauen (Kanada; 18–45 a; 117)	0,21	0,52	2,4^g)^	< BG–26,3 (DF = 75)	Siddique et al. 2020
Erwachsene (Vietnam; 18–76 a; 42)	0,02	0,105	11,9^g)^ 2,65 (GM)^g)^	0,03–49,6 (DF = 50)	Trinh et al. 2024
Erwachsene (Belgien; Ø 40,8 a; 59)	–	0,52	–	< NWG–15 (DF = 15)	Van den Eede et al. 2013 b
Erwachsene (USA; 33,8 ± 12 a; 213 Proben von 19 Personen)	0,0102	–	0,737 0,414 (GM) 0,359 (Median)	0,021–5,650 (DF = 100)	Wang et al. 2019
Erwachsene (China; 44,62 ± 10,58 a; 1981)	0,064	–	0,12 (GM)^d)^ 0,12 (Median)^d)^	< BG–0,22 (DF = 60,9)	Xu et al. 2024

a: Jahr; BG: Bestimmungsgrenze; DF (*detection frequency*): Nachweishäufigkeit in % (Prozentsatz der Messwerte oberhalb der Nachweis- bzw. Bestimmungsgrenze); GM: geometrischer Mittelwert; k. A.: keine Angabe; n: Probenanzahl; NWG: Nachweisgrenze

a) Spontanurin (n = 309)

b) 24-h-Urin (n = 10)

c) gemessen als Duplikate, die in Glas- und Kunststoffflaschen aufbewahrt wurden

d) Die zusammenfassenden Statistiken wurden unter Verwendung der NWG/√2 für Werte unterhalb der NWG berechnet.

e) gemessen als Duplikate, die nur in Kunststoff-Lagerungsgefäßen aufbewahrt wurden

f) 95- %‑Konfidenzintervall

g) Die zusammenfassenden Statistiken wurden unter Verwendung der NWG/2 für Werte unterhalb der NWG berechnet.

**Tab.2:** BDCPP im Urin von potenziell exponierten Arbeitern

Studienkollektiv (Land; Alter; n)	NWG [μg/l]	BG [μg/l]	BDCPP [μg/l]		Literatur
			Mittelwert ± Standardabweichung	Bereich	
Feuerwehrfrauen (USA; ≥ 18 a; 86)	0,2	–	4,08 (GM), 4,53 (GSD)^a)^	1,30–32,22 (DF = 100)	Trowbridge et al. 2022
Büroangestellte (USA; ≥ 18 a; 84)			0,96 (GM), 3,99 (GSD)^a)^	< NWG–8,72 (DF = 90)	
Erwachsene Feuerwehrleute (USA; Alter k. A.; 146)	0,11	–	3,4 (Median)	0,30–44 (DF = 100)	Jayatilaka et al. 2017
Kontrollen, Allgemeinbevölkerung (USA; Alter k. A.; 76)			0,69 (Median)	0,31–6,8 (DF = 100)	
Feuerwehrmänner (Südkorea; 38–56 a; 149)	–	0,14	0,12^a)^ < BG (Median)	< NWG–2,40^a)^ (DF = 6,7)	Lim et al. 2023
Feuerwehrleute (USA; 21–52 a; 36)	0,11	–	2,38 μg/g Krea (GM), 2,12 (GSD); vor dem Feuer^a)^	(DF = 100)	Mayer et al. 2021
Arbeiter bei der Demontage von Elektroschrott (China; 18–72 a; 30); Morgenurin	–	0,20	0,58 (Median)	< NWG–18,5 (DF = 80)	Qin et al. 2021
Arbeiter bei der Demontage von Elektroschrott (China; 18–72 a; 30); Abendurin			0,53 (Median)	< NWG–20,4 (DF = 93)	
Arbeiter im Elektroschrott-Recycling (China; 29–64 a; 42): Morgenurin an Tag 3	–	0,02	1,66 ± 2,78 0,89 (Median)	< NWG–12,8 (DF = 91)	Shi et al. 2019
Arbeiter im Elektroschrott-Recycling (China; 29–64 a; 42); Abendurin an Tag 3			1,03 ± 1,13 0,53 (Median)	< NWG–3,91 (DF = 87)	
Hotelangestellte (China; 23–57 a; 26)	0,064	0,21	0,19 (GM); 0,21 (Median)	< BG–2,1 (DF = 79)	Tao et al. 2018
Arbeiter in der Müllverbrennung (China; 28,2 ± 4,3 a; 73)	0,12	–	0,18 (Median)^b)^	< NWG–1,75 (DF = 82,2)	Wu et al. 2023
Kontrollen, Allgemeinbevölkerung (China; 27,4 ± 5,1 a; 97)			0,06 (Median)^b)^	< NWG–0,61 (DF = 11,3)	
Arbeiter im Elektroschrott-Recycling (China; k. A.; 88)	–	0,03	0,23 (Median)^b)^	< NWG–31,8 (DF = 82)	Yan et al. 2018
Arbeiter in der Müllverbrennung (China; k. A.; 30)			0,22 (Median)^b)^	< NWG–3,56 (DF = 93)	

a: Jahr; BG: Bestimmungsgrenze; DF (*detection frequency*): Nachweishäufigkeit in % (Prozentsatz der Messwerte oberhalb der Nachweis- oder Bestimmungsgrenze); GM: geometrischer Mittelwert; GSD: geometrische Standardabweichung; k. A.: keine Angabe; n: Probenanzahl; NWG: Nachweisgrenze

a) Die zusammenfassenden Statistiken wurden unter Verwendung von NWG/√2 für Werte unterhalb der NWG berechnet.

b) Die zusammenfassenden Statistiken wurden unter Verwendung von NWG/2 für Werte unterhalb der NWG berechnet.

## 3 Grundlage des Verfahrens

Für die Bestimmung von BDCPP in Urin werden die verdünnten Urinproben leicht angesäuert und mit dem internen Standard d_10_‑BDCPP versetzt. Anschließend werden die Proben durch Festphasenextraktion unter Verwendung von SPE-Kartuschen (schwacher Anionenaustauscher, Polymer) aufgereinigt. BDCPP wird flüssigkeitschromatographisch von Begleitkomponenten getrennt und anschließend mittels Tandem-Massenspektrometrie unter simultaner Verwendung von chemischer Ionisierung bei Atmosphärendruck (APCI) und Elektrospray-Ionisierung (ESI) detektiert. Die quantitative Auswertung erfolgt mittels externer Kalibrierung.

## 4 Geräte, Chemikalien und Lösungen

### 4.1 Geräte

- LC‑MS/MS‑System (z. B. Agilent 6460 Triple-Quad mit einer Multimode-Quelle gekoppelt an ein Agilent 1200 HPLC-System, Agilent Technologies, Inc., Santa Clara, CA, USA)
- Kinetex^®^ LC-Säule (2,6 μm Biphenyl 100 Å, 100 × 2,1 mm) (z. B. Nr. 00D‑4622‑AN, Phenomenex Inc., Torrance, CA, USA) mit einer UHPLC-Biphenyl-Vorsäule mit 2,1 mm ID (z. B. SecurityGuard ULTRA Cartridge Nr. AJ0‑9209, Phenomenex Inc., Torrance, CA, USA)
- Analysenwaage (z. B. Sartorius AG, Göttingen)
- SPE-Vakuumstation (z. B. IST VacMaster, Biotage Sweden AB, Uppsala, Schweden)
- Abblasstation (z. B. TurboVap^®^ LV, Biotage Sweden AB, Uppsala, Schweden)
- SPE-Kartuschen (schwacher Anionenaustauscher, Polymer) (z. B. Nr. 8B‑S038‑UBJ, Strata‑X‑AW 33 μm, 60 mg/3 ml, Phenomenex Inc., Torrance, CA, USA)
- pH‑Indikatorstreifen pH 0–14 (z. B. Nr. 1.09535, MQuant^®^, Merck KGaA, Darmstadt)
- pH‑Indikatorstreifen pH 5–10 (z. B. Nr. 1.09533, MQuant^®^, Merck KGaA, Darmstadt)
- Spritzenvorsatzfilter (Nylonmembran, Membrandurchmesser 13 mm, Porengröße 0,2 μm) (z. B. Nr. 6870‑1302, Whatman^®^ GD/X^TM^, GE HealthCare Life Sciences, Buckinghamshire, Vereinigtes Königreich)
- 10‑ml-, 100‑ml- und 1000‑ml-Messkolben (z. B. BRAND GMBH + CO KG, Wertheim)
- 100‑ml-Weithalsbraunglasflaschen mit Polypropylen-Schraubverschluss als Urinsammelgefäße (z. B. Nr. 215‑4382, VWR International GmbH, Darmstadt)
- 100‑ml-Messzylinder (z. B. SCHOTT AG, Mainz)
- 15‑ml-Polypropylenröhrchen mit Schraubverschluss (z. B. Nr. 525‑0604, VWR International GmbH, Darmstadt)
- 8‑ml‑Gewindefläschchen (z. B. Nr. 548‑0821A, VWR International GmbH, Darmstadt)
- 2‑ml‑Schraubgläser und passende Schraubkappen mit PTFE/Silikon‑Septen (z. B. Nr. 5182‑0715 und Nr. 5185‑5820, Agilent Technologies Netherlands BV, Amstelveen, Niederlande)

### 4.2 Chemikalien

Wenn nicht anders angegeben, sind alle genannten Chemikalien mindestens in p. a.‑Qualität zu verwenden.

- BDCPP (z. B. Nr. TRC‑B419095‑10MG, Toronto Research Chemicals, Toronto, Kanada)
- d_10_‑BDCPP (ISTD) (z. B. Nr. TRC‑B419097‑10MG, Toronto Research Chemicals, Toronto, Kanada)
- Essigsäure, ≥ 99,7 % (z. B. Nr. 695092, Fluka^TM^, Honeywell Deutschland Holding GmbH, Offenbach)
- Methanol, ≥ 99,8 %, HiPerSolv CHROMANORM^®^ für HPLC (z. B. Nr. 152505N, VWR International BV, Amsterdam, Niederlande)
- Acetonitril, Ultra-Gradient, ultrarein (z. B. Nr. 10614471, J. T. Baker Chemicals N. V., Deventer, Niederlande)
- Pyrrolidin (z. B. Nr. 807494, Merck KGaA, Darmstadt)
- Ameisensäure (z. B. Nr. 56302, Honeywell Specialty Chemicals Seelze GmbH, Seelze)
- Hochreines Wasser (Widerstand von 18,2 MΩ × cm) (z. B. aus einer zentralen Versorgungsanlage)
- Stickstoff 5.0 (z. B. Air Liquide Deutschland GmbH, Düsseldorf)

### 4.3 Lösungen

- Methanol : Wasser (1 : 4, V/V)
  In einem 100‑ml-Messkolben werden 20 ml Methanol mit 80 ml hochreinem Wasser gemischt.
- 5% Pyrrolidin in Acetonitril
  5 ml Pyrrolidin werden in einen 100‑ml-Messkolben pipettiert. Der Kolben wird anschließend mit Acetonitril bis zur Markierung aufgefüllt.
- Essigsäure (0,1 mol/l)
  572 μl Essigsäure werden in einen 100-ml-Messkolben, in dem hochreines Wasser vorgelegt ist, pipettiert. Anschließend wird der Kolben mit hochreinem Wasser bis zur Markierung aufgefüllt.
- Laufmittel A (0,1 % (V/V) Ameisensäure in hochreinem Wasser)
  1 ml der konzentrierten Ameisensäure wird in einen 1000‑ml-Messkolben pipettiert. Anschließend wird der Kolben bis zur Markierung mit hochreinem Wasser aufgefüllt.
- Laufmittel B (0,1 % (V/V) Ameisensäure in Methanol)
  1 ml der konzentrierten Ameisensäure wird in einen 1000‑ml-Messkolben pipettiert. Anschließend wird der Kolben bis zur Markierung mit Methanol aufgefüllt.

### 4.4 Interner Standard (ISTD)

- ISTD‑Stammlösung (1000 mg/l)
  In einen 10‑ml-Messkolben werden 10 mg des ISTD d_10_‑BDCPP genau eingewogen und in etwas Acetonitril gelöst. Der Messkolben wird anschließend mit Acetonitril bis zur Markierung aufgefüllt.
- ISTD‑Arbeitslösung (10 mg/l)
  100 μl der ISTD‑Stammlösung werden in einen 10‑ml-Messkolben pipettiert. Anschließend wird der Messkolben bis zur Markierung mit Methanol aufgefüllt.
- ISTD-Dotierlösung (1 mg/l)
  100 μl der ISTD‑Arbeitslösung werden in ein 2‑ml-Gläschen pipettiert. Anschließend werden 900 μl Methanol hinzugefügt und die Lösung wird gemischt.

Die ISTD‑Stamm- und die ISTD-Arbeitslösung werden im Gefrierschrank bei −21 °C gelagert. Die ISTD-Dotierlösung wird stets frisch angesetzt.

### 4.5 Kalibrierstandards

- BDCPP‑Stammlösung (1000 mg/l)
  In einen 10‑ml-Messkolben werden etwa 10 mg BDCPP exakt eingewogen und in etwas Acetonitril gelöst. Der Messkolben wird anschließend mit Acetonitril bis zur Markierung aufgefüllt.
- BDCPP‑Arbeitslösung (10 mg/l)
  100 μl der BDCPP‑Stammlösung werden in einen 10‑ml-Messkolben pipettiert. Anschließend wird der Messkolben bis zur Markierung mit Methanol aufgefüllt.
- BDCPP‑Dotierlösung 1 (DL 1; 1 mg/l)
  Je 100 μl der BDCPP‑Arbeitslösung und der d_10_‑BDCPP‑Arbeitslösung werden in ein 2‑ml-Gläschen pipettiert. Anschließend werden 800 μl Methanol zugegeben und die Lösung wird gemischt.
- BDCPP‑Dotierlösung 2 (DL 2; 0,01 mg/l)
  10 μl DL 1 und 990 μl Methanol werden in ein 2‑ml-Gläschen zusammenpipettiert und gemischt.

Die BDCPP‑Stamm- und die BDCPP-Arbeitslösung werden im Gefrierschrank bei −21 °C gelagert. Die BDCPP‑Dotier­lösungen werden jeweils frisch angesetzt.

Die Kalibrierstandards werden gemäß dem in Tabelle 3 angegebenen Pipettierschema in 2‑ml-Gläschen hergestellt. Die Konzentration des ISTD entspricht dabei immer der BDCPP-Konzentration des Kalibrierstandards. Diese Kalibrierstandards werden analog zu den Urinproben gemäß Abschnitt 5.2 ohne Zugabe von weiterem ISTD aufbereitet und anschließend analysiert.

**Tab.3:** Pipettierschema für die Herstellung der Kalibrierlösungen für die Bestimmung von BDCPP im Urin

Kalibrierstandard	DL 2 [μl]	DL 1 [μl]	Methanol : Wasser (1 : 4, V/V) [μl]	BDCPP [μg/l]	d_10_‑BDCPP [μg/l]
1	10	–	990	0,1	0,1
2	20	–	980	0,2	0,2
3	50	–	950	0,5	0,5
4	100	–	900	1	1
5	200	–	800	2	2
6	500	–	500	5	5
7	–	10	990	10	10
8	–	20	980	20	20
9	–	50	950	50	50
10	–	100	900	100	100

## 5 Probenahme und Probenaufbereitung

### 5.1 Probenahme

Die Urinproben werden in vorgereinigten 100‑ml-Probengläsern aus Braunglas gesammelt. Wenn die Proben nicht ­sofort aufgearbeitet werden, müssen sie während des Transports gekühlt und bis zur Analyse bei −21 °C gelagert werden. Unter diesen Bedingungen sind die Urinproben mindestens eine Woche lang stabil (siehe Abschnitt 11.5).

### 5.2 Probenaufbereitung

Eingefrorene Urinproben werden bei Raumtemperatur aufgetaut und anschließend gut gemischt. 5 ml Urin werden in ein 15‑ml-Polypropylenröhrchen pipettiert und 10 μl der ISTD‑Dotierlösung (1 mg d_10_‑BDCPP/l) zugesetzt. Nach einer 1 ∶ 1 (V/V) Verdünnung mit hochreinem Wasser wird der pH-Wert mit etwa 450 μl verdünnter Essigsäure (0,1 mol/l) auf pH 6 eingestellt. Der pH‑Bereich wird zunächst mit pH 0–14-Teststreifen überprüft und eine endgültige Kontrolle der korrekten Einstellung mit pH 5–10-Teststreifen vorgenommen.

Anschließend wird eine Festphasenextraktion (SPE) unter Verwendung von Phenomenex StrataX AW‑Kartuschen durchgeführt. Dazu werden die Kartuschen mit 2 ml Methanol, gefolgt von 2 ml hochreinem Wasser, konditioniert. Dann werden die Kartuschen mit den Urinproben beladen (< 1 ml/min), mit 2 ml hochreinem Wasser gewaschen und unter Vakuum getrocknet. Der Analyt wird mit 2 ml Acetonitril, das 5 % Pyrrolidin enthält, von den Kartuschen in 8‑ml-Glasfläschchen eluiert. Die eluierten Proben werden im Stickstoffstrom bei Raumtemperatur zur Trockene abgeblasen. Schließlich wird der Rückstand in 500 μl Methanol ∶ Wasser (1 ∶ 4, V/V) aufgenommen und durch einen 0,2‑μm‑Spritzenvorsatzfilter in ein 2‑ml-HPLC-Vial filtriert.

## 6 Instrumentelle Arbeitsbedingungen

Die analytische Bestimmung erfolgte an einer Gerätekonfiguration bestehend aus einer LC‑Anlage und einem Tandem-Massenspektrometer (LC‑MS/MS). Die Entwickler der Methode verwendeten ein Tandem-Massenspektrometer mit Multimode-Quelle (Agilent 6460) und setzten gleichzeitig chemische Ionisierung bei Atmosphärendruck und Elektrospray-Ionisierung ein. 

Die nachfolgend beschriebenen Einstellungen sind gerätespezifisch und müssen vom Anwender geprüft und gege­benenfalls angepasst werden. Die Angaben sollen daher nur als Orientierungshilfe dienen. Gegebenenfalls sind an Geräten anderer Hersteller auch zusätzliche Einstellungen notwendig.

### 6.1 Flüssigkeitschromatographie

**Table:** 

Analytische Säule:	Kinetex^®^ core-shell silica (2,6 μm Biphenyl 100 Å, 100 × 2,1 mm)
Vorsäule:	UHPLC Biphenyl, 2,1 mm ID
Trennprinzip:	*Reversed Phase* (Umkehrphase)
Temperatur Säulenofen:	60 °C
Injektionsvolumen:	5 μl
Laufmittel:	A: 0,1 % Ameisensäure in hochreinem Wasser
	B: 0,1 % Ameisensäure in Methanol
Laufzeit:	15 min
Gradientenprogramm:	siehe Tabelle 4

**Tab.4:** Gradientenprogramm für die Bestimmung von BDCPP in Urin

Zeit [min]	Laufmittel A [%]	Laufmittel B [%]	Flussrate [ml/min]	Druck^a)^ [hPa]
0,0	90	10	0,500	600 000
10,0	10	90		
13,0	10	90		
13,1	90	10		
15,0	90	10		

a) Anfangsdruck: 330 000 hPa bei 90 % Laufmittel A

### 6.2 Tandem-Massenspektrometrie

**Table:** 

Ionisierung:	Gleichzeitige chemische Ionisierung bei Atmosphärendruck (APCI) und Elektrospray-Ionisierung (ESI) im negativen Ionisierungsmodus
Detektionsmodus:	*Multiple Reaction Monitoring* (MRM)
Gastemperatur (N_2_):	350 °C
Temperatur des Verdampfers:	150 °C
Gasfluss (N_2_):	5 ml/min
Verneblerdruck:	60 psi
Kapillarspannung:	−4000 V
Koronastrom:	1 μA
Multiplier:	500 delta EMV (+)
Parameterspezifische Einstellungen:	siehe Tabelle 5

Die Retentionszeiten, Massenübergänge und weitere MS/MS‑Parameter sind in Tabelle 5 aufgeführt. Um Signalsuppression zu reduzieren werden alle Urinproben vor der Messung 1 : 5 verdünnt.

**Tab.5:** Retentionszeiten, Massenübergänge und MS/MS‑Parameter für die Bestimmung von BDCPP in Urin

Substanz	Retentionszeit [min]	Vorläufer-Ion (*m/z*)	Produkt-Ion (*m/z*)	Kollisionsenergie [V]	Cone-Spannung [V]	Beschleunigungsspannung [V]
BDCPP	5,97	319	35,1^a)^	16	60	5
		319	37,0^b)^	16	60	5
		318	35,1^b)^	8	80	5
d_10_‑BDCPP	5,89	329	35,1^a)^	12	80	5
		329	37,1^b)^	16	80	5
		328	35,0^b)^	8	80	5

a) Quantifier

b) Qualifier

## 7 Analytische Bestimmung

Zur analytischen Bestimmung von BDCPP werden jeweils 5 μl der nach Abschnitt 5.2 aufgearbeiteten Urinproben in das LC‑MS/MS-System injiziert und unter den in Abschnitt 6 angegebenen Bedingungen analysiert. Die Identifizierung des Analyten BDCPP erfolgt anhand der Retentionszeit und der charakteristischen Massenübergänge.

Die in Tabelle 5 angegebenen Retentionszeiten können nur als Anhaltspunkt dienen. Der Anwender der Methode muss sich von der Trennleistung der verwendeten Säule und dem daraus resultierenden Retentionsverhalten des Analyten überzeugen.

**Abb.2:**
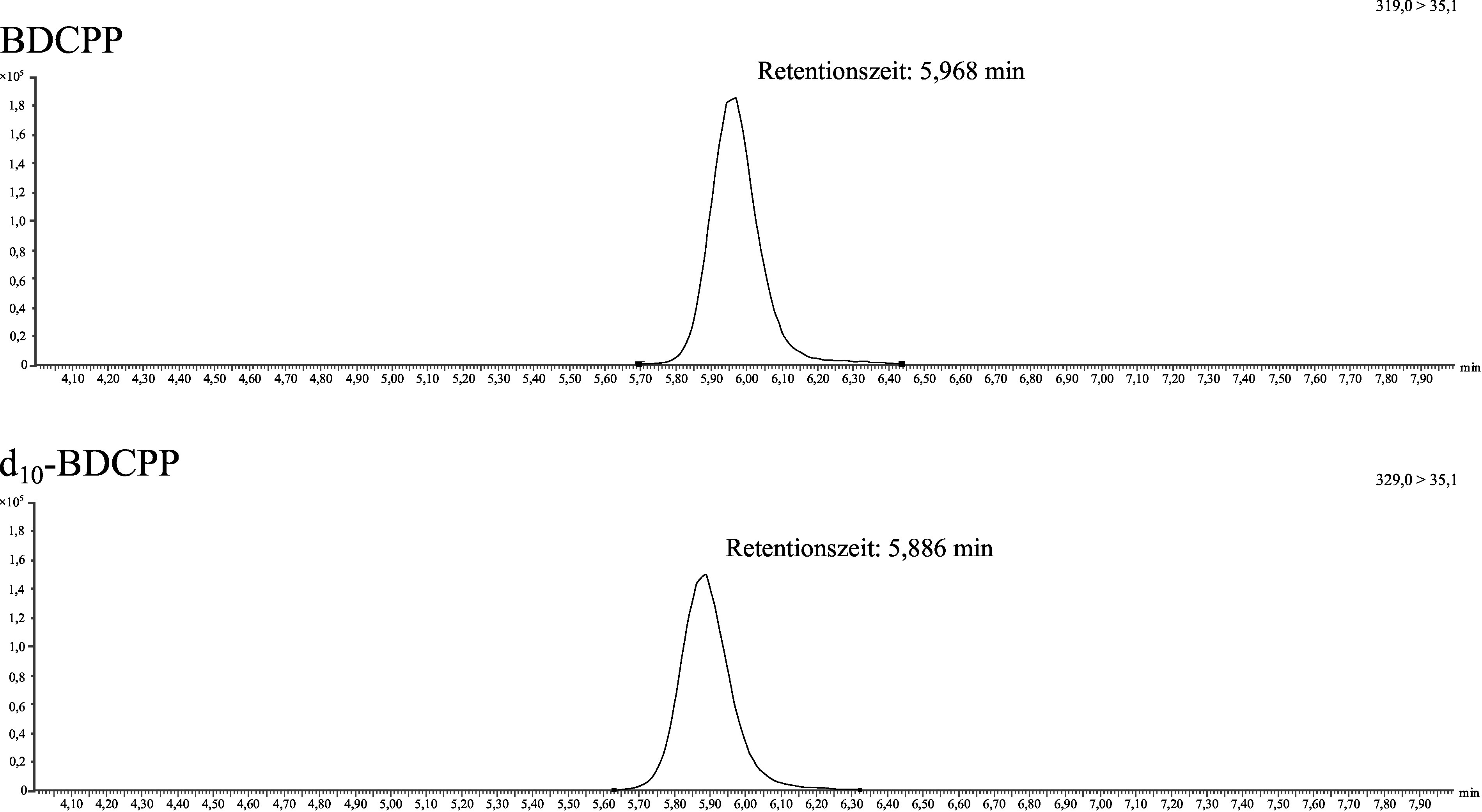
Chromatogramm eines Standards, der sowohl mit 90 μg BDCPP als auch mit 90 μg d_10_‑BDCPP pro Liter dotiert war

**Abb.3:**
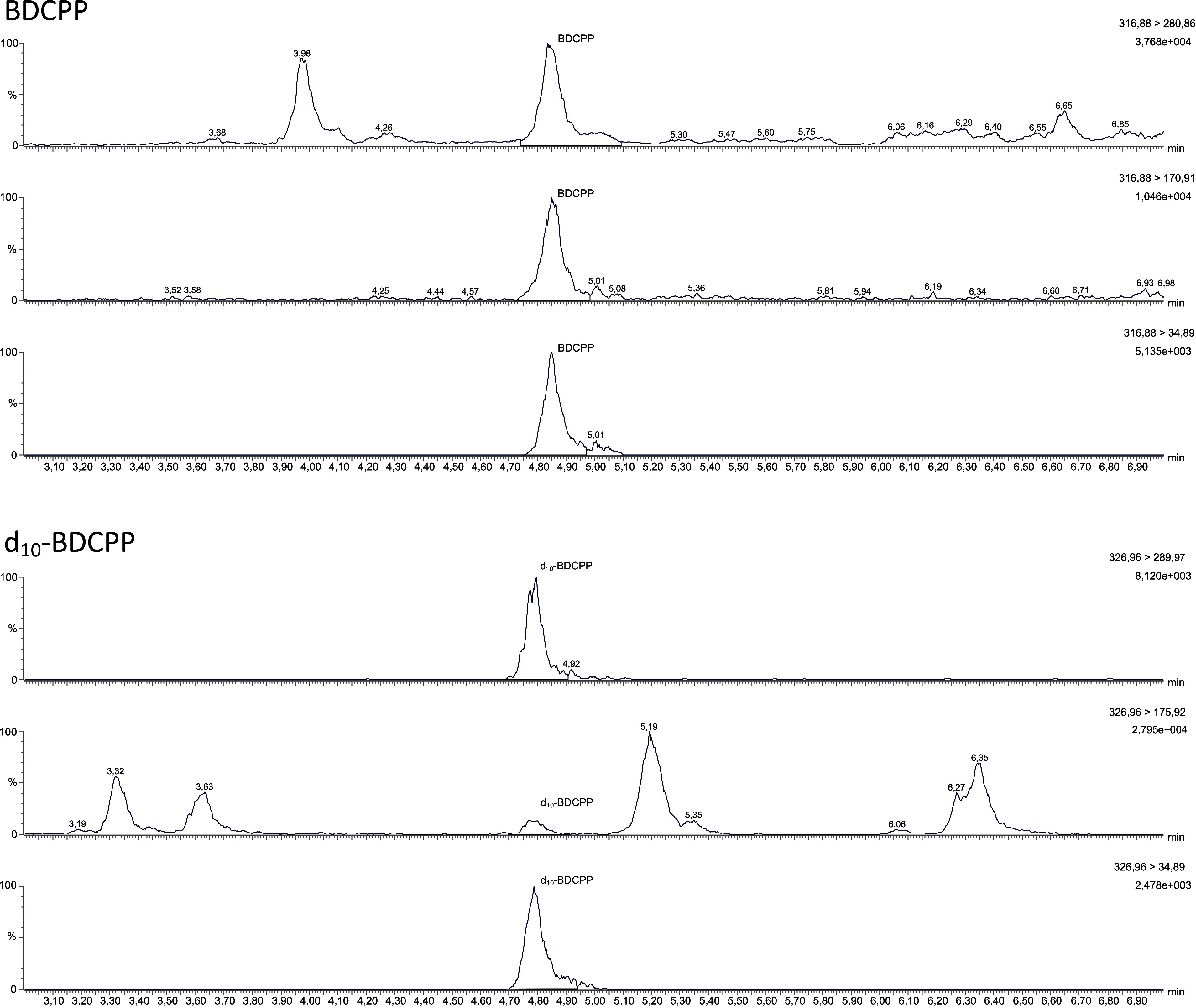
Chromatogramm eines mit 20 μg BDCPP sowie 10 µg d_10_-BDCPP pro Liter dotierten Poolurins

Abbildung 2 zeigt ein repräsentatives Chromatogramm eines mit 90 μg BDCPP sowie mit 90 μg d_10_‑BDCPP pro Liter dotierten Standards (Methodenentwicklung). Abbildung 3 zeigt ein repräsentatives Chromatogramm eines mit 20 μg BDCPP sowie mit 10 μg d_10_‑BDCPP pro Liter dotierten Poolurins (Methodenprüfung).

## 8 Kalibrierung

Die Kalibrierlösungen werden wie in Abschnitt 4.5 beschrieben hergestellt, analog zu den Urinproben ohne ­weitere Zugabe von ISTD (siehe Abschnitt 5.2) aufgearbeitet und analysiert. Die Kalibriergerade wird erstellt, indem die Peakflächen des Analyten bzw. des ISTD gegen die dotierten Konzentrationen aufgetragen werden. Die Kalibriergerade des d_10_‑BDCPP wird zur Berechnung der Wiederfindung des ISTD verwendet (siehe Abschnitt 9*)*.

**Abb.4:**
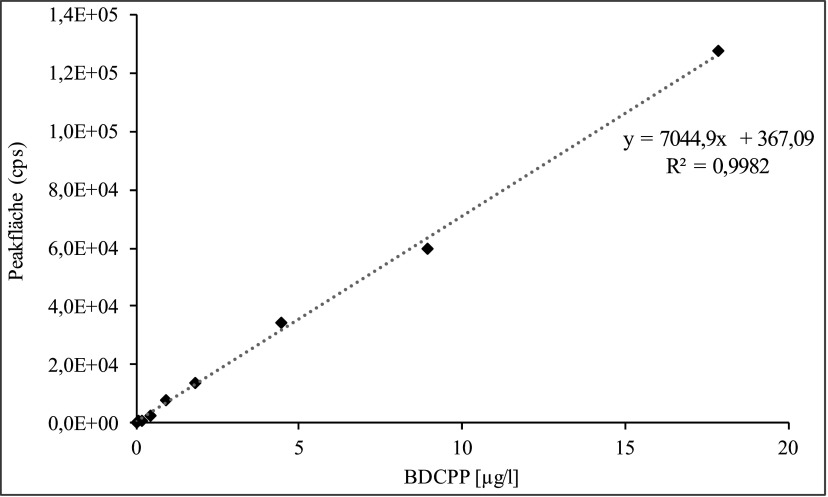
Kalibriergerade für die Bestimmung von BDCPP im Urin der beruflich nicht belasteten Allgemeinbevölkerung

Die Kalibriergerade von BDCPP verläuft bis zu einer Konzentration von 1000 μg/l linear. Für den Nachweis einer beruflichen Exposition sollte ein Konzentrationsbereich von 0,1–100 µg/l ausreichend sein. Für die Quantifizierung der Hintergrundbelastung in der nicht beruflich belasteten Allgemeinbevölkerung wird ein Kalibrierbereich von 0,1–20 μg/l verwendet (siehe Abbildung 4). 

## 9 Berechnung der Analysenergebnisse

Die Steigung der Kalibriergerade wird durch lineare Regression berechnet und zur Quantifizierung verwendet. Dazu wird von der achsenabschnittskorrigierten BDCPP-Peakfläche der Probe die achsenabschnittskorrigierte Peakfläche des Blindwerts abgezogen und unter Berücksichtigung der Kalibriergeradensteigung der Analytgehalt in μg/l berech­net. Zudem werden bei der Konzentrationsberechnung die unterschiedlichen Volumina der Kalibrierstandards und der gemessenen Urinproben berücksichtigt. 

Die Prüfer der Methode verwendeten in Urin angesetzte Kalibrierstandards, die zunächst nur BDCPP enthielten. Von diesen wurden 5 ml analog zu den Urinproben aufgearbeitet und enthielten dadurch eine stets gleiche Konzentration von 10 μg ISTD pro Liter Urin. 

**Abb.5:**
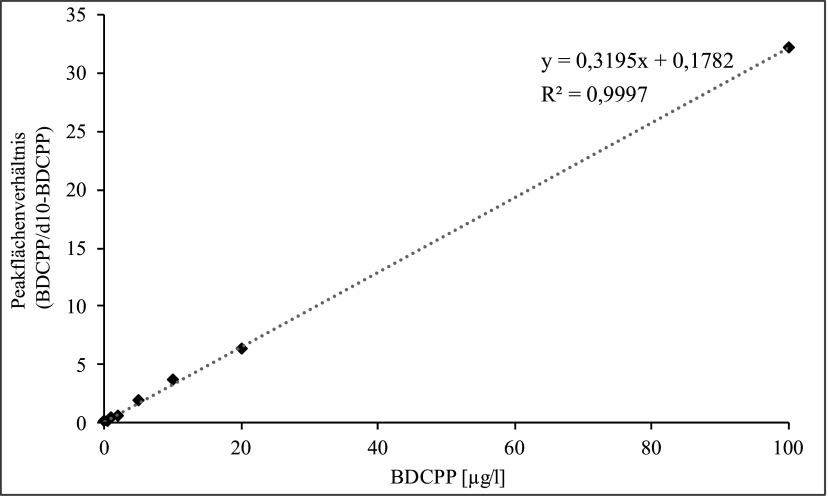
Kalibriergerade für die Bestimmung von BDCPP in Urin (Methodenprüfung)

Während der Methodenprüfung wurde die Kalibriergerade erstellt, indem die Peakflächenverhältnisse von Analyt und ISTD gegen die entsprechenden BDCPP-Konzentrationen aufgetragen wurden (siehe Abbildung 5). Falls Leerwerte auftraten, wurden diese durch Subtraktion von allen Messpunkten berücksichtigt. Zur Berechnung der BDCPP-Konzentration in einer zu messenden Urinprobe wurde die für BDCPP ermittelte Peakfläche durch die Peakfläche des deuterierten ISTD geteilt. Mit dem so erhaltenen Quotienten wurde über die Kalibrierfunktion das Analysenergeb­nis in μg/l berechnet.

Dieser Ansatz ist mathematisch identisch mit dem von den Entwicklern der Methode gewählten Ansatz.

## 10 Standardisierung der Messergebnisse und Qualitätssicherung

Zur Sicherung der Qualität der Analysenergebnisse wird gemäß den Richtlinien der Bundesärztekammer und den Angaben in dem von der Kommission veröffentlichten allgemeinen Kapitel verfahren (Bader et al. 2010; Bundesärztekammer 2014).

Zur Qualitätssicherung der einzelnen Analyseläufe werden parallel zu den Proben mindestens drei Urine mit bekan­n­ten Analytkonzentrationen aufgearbeitet und analysiert. Da derzeit keine Kontrollmaterialien für BDCPP kommerziell erhältlich sind, müssen diese im eigenen Labor hergestellt werden. Dazu wird gepoolter Urin von nicht beruflich gegen TDCPP exponierten Personen verwendet und mit BDCPP in Konzentrationen von z. B. 1,0 μg, 10 μg sowie 100 μg pro Liter versetzt.

Gleichzeitig werden bei jedem Analyselauf Leerwerte (hochreines Wasser) und Reagenzienleerwerte mitgemessen, um mögliche Störungen durch Reagenzien, Matrixkomponenten oder den ISTD zu erkennen.

## 11 Beurteilung des Verfahrens

Die Zuverlässigkeit des Verfahrens wurde durch eine umfassende Validierung sowie durch Nachstellung und Prüfung der Methode in einem zweiten, unabhängigen Labor bestätigt.

### 11.1 Präzision

#### Präzision in der Serie

Zur Bestimmung der Präzision in der Serie wurde eine Poolurinprobe mit 0,9 μg, 9 μg oder 90 μg BDCPP/l dotiert, achtfach parallel aufgearbeitet und analysiert. Die aus den Messergebnissen berechneten Präzisionsdaten sind Tabelle 6 zu entnehmen.

**Tab.6:** Präzision in der Serie für die Bestimmung von BDCPP in Urin (n = 8)

Analyt	Dotierte Konzentration [μg/l]	Standardabweichung (rel.) *s_w_* [%]	Streubereich *u* [%]
BDCPP	0,9	6,5	15,4
	9	3,1	7,3
	90	5,5	13,0

#### Präzision von Tag zu Tag

Für die Präzision von Tag zu Tag wurde das gleiche Material wie für die Präzision in der Serie verwendet. Die Proben wurden an acht Tagen aufgearbeitet und analysiert. Die so erhaltenen Präzisionsdaten sind in Tabelle 7 aufgeführt.

**Tab.7:** Präzision von Tag zu Tag für die Bestimmung von BDCPP in Urin (n = 8)

Analyt	Dotierte Konzentration [μg/l]	Standardabweichung (rel.) *s_w_* [%]	Streubereich *u * [%]
BDCPP	0,9	8,5	20,1
	9	8,7	20,6
	90	5,5	13,0

### 11.2 Richtigkeit

Die relative Wiederfindung wurde anhand der Daten zur Präzision von Tag zu Tag berechnet, indem die gemessenen Konzentrationen zu den jeweils dotierten Konzentrationen ins Verhältnis gesetzt wurden. Die Ergebnisse sind in Tabelle 8 dargestellt.

**Tab.8:** Relative Wiederfindung für die Bestimmung von BDCPP im Urin (n = 8)

Analyt	Dotierte Konzentration [μg/l]	Mittlere relative Wiederfindung *r * [%]
BDCPP	0,9	93,6
	9	99,5
	90	100,5

### 11.3 Einfluss verschiedener Urinmatrices

Der Einfluss verschiedener Urinmatrices auf Präzision und Wiederfindung wurde an zehn individuellen Urinproben untersucht. Die Kreatiningehalte der Urinproben lagen zwischen 1,3 mmol und 22,7 mmol pro Liter. Die Proben wurden mit 90 μg BDCPP pro Liter Urin dotiert und anschließend aufgearbeitet und analysiert.

Die aus den Messergebnissen errechnete Standardabweichung und die mittlere relative Wiederfindung sind in Tabelle 9 aufgeführt. Es ist ersichtlich, dass die individuelle Urinmatrix keinen Einfluss auf die Präzision und Richtigkeit der Analysenergebnisse hat.

**Tab.9:** Einfluss verschiedener Urinmatrices auf die Bestimmung von BDCPP im Urin (n = 10)

Analyt	Dotierte Konzentration [μg/l]	Standardabweichung (rel.) *s*_w_ [%]	Relative Wiederfindung *r* [%]
BDCPP	90,0	5,8	92,3

### 11.4 Nachweisgrenze und Bestimmungsgrenze

Die Nachweis- und die Bestimmungsgrenze für die Bestimmung von BDCPP in Urin wurden gemäß NEN 7777+C1 (NEN 2012) ermittelt (Mehrfachbestimmungen an einer Laborprobe). Zu diesem Zweck wurden acht Urinproben mit 0,9 μg BDCPP pro Liter Urin dotiert und an einem Tag aufgearbeitet und analysiert. Zudem wurden die aufgearbeiteten Proben an acht verschiedenen Tagen analysiert. Die berechneten BDCPP-Konzentrationen lagen im Bereich von 0,73–1,00 μg pro Liter, für die weiteren Berechnungen wurde der Mittelwert von 0,84 μg BDCPP/l verwendet.

Die Nachweisgrenze wurde als das Dreifache, die Bestimmungsgrenze als das Zehnfache der Standardabweichung vom Mittelwert berechnet. Tabelle 10 zeigt die entsprechenden Werte für die Bestimmung von BDCPP im Urin.

**Tab.10:** Nachweis- und Bestimmungsgrenze für die Bestimmung von BDCPP in Urin

Analyt	Nachweisgrenze [μg/l]	Bestimmungsgrenze [μg/l]
BDCPP	0,06	0,2

### 11.5 Stabilität von BDCPP im Urin und in den Extrakten

Zur Beurteilung der Lagerstabilität von BDCPP in den Urinproben und in den Extrakten wurden Lagerungsversuche durchgeführt. Zu diesem Zweck wurde gepoolter Urin aliquotiert und mit 0,9 μg, 9 μg und 90 μg BDCPP pro Liter ­dotiert und bei verschiedenen Temperaturen (−21 °C, 3 °C und 21 °C) im Dunkeln gelagert. Die Proben wurden nach 0, 24, 48, 72 und 168 Stunden aufgearbeitet und analysiert. Zusätzlich wurden SPE-Extrakte direkt aufgearbeiteter Proben bei −21 °C, 3 °C und 21 °C gelagert und nach den oben genannten Lagerzeiten analysiert.

Mit relativen Wiederfindungen zwischen 91 % und 104 % zeigten die Lagerungsversuche, dass BDCPP unter den getes­teten Bedingungen stabil ist.

### 11.6 Störeinflüsse

Mögliche Carry‑Over-Effekte wurden untersucht, indem aufgearbeitete Proben eines mit 90 μg BDCPP pro Liter dotierten Urins und eines undotierten Urins abwechselnd vermessen wurden (jeweils n = 6). Es trat kein Carry‑Over-Effekt auf, da sich in keiner der sechs undotierten Urinproben BDCPP nachweisen ließ.

Für die Methodenentwicklung wurde ein LC‑MS/MS-System mit einer Agilent Multimode-Quelle verwendet, die eine simultane ESI- und APCI-Ionisierung ermöglicht. Die Empfindlichkeit der Mixed‑Mode-Einstellung ist im Vergleich mit den einzelnen Ionenquellen ähnlich oder besser (Fischer und Perkins 2005).

Das Gerät der Methodenprüfer verfügte nicht über eine solche Multimode-Quelle, so dass die Prüfer zwischen dem reinen ESI- und dem reinen APCI-Modus wählen mussten. Da die Ionisierungsrate bei der ESI-Ionisierung besser war als bei der APCI-Ionisierung, wurde der ESI-Modus für die Methodenprüfung verwendet. Bei ausschließlicher Verwendung von ESI konnten die Prüfer die von den Methodenentwicklern angegebenen Massenübergänge bestätigen, aber andere Massenübergänge erwiesen sich als intensiver. Die alternativen Massenübergänge, die bei der Prüfung der Methode verwendet wurden, sind in Tabelle 11 zusammen mit den Retentionszeiten und weiteren MS/MS-Parametern aufgeführt.

Die von den Prüfern der Methode ermittelte Bestimmungsgrenze (3,0 μg/l) war höher als die von den Entwicklern angegebene (0,2 μg/l) und damit zu hoch für die Bestimmung der meisten Hintergrundbelastungen. Die gleichzeitige ESI- und APCI-Ionisierung ist somit für eine empfindliche Quantifizierung der BDCPP-Hintergrundgehalte mit dieser Methode unerlässlich.

**Tab.11:** Retentionszeiten, Massenübergänge und MS/MS-Parameter für die Bestimmung von BDCPP in Urin (Methodenprüfung)

Substanz	Retentionszeit [min]	Vorläufer-Ion (*m/z *)	Produkt-Ion (*m/z *)	Kollisionsenergie [V]	Cone-Spannung [V]
BDCPP	4,6	316,9	280,9^a)^	6	14
		316,9	170,9^b)^	8	14
		316,9	34,9^b)^	6	14
d_10_‑BDCPP	4,5	327,0	290,0^a)^	6	8
		327,0	175,9^b)^	8	8
		327,0	34,9^b)^	6	8

a) Quantifier

b) Qualifier

## 12 Diskussion der Methode

Die hier beschriebene LC‑MS/MS-Methode basiert auf grundlegenden Verfahrensschritten aus veröffentlichten Metho­den zur Quantifizierung von BDCPP in Urin (Carignan et al. 2013; Cooper et al. 2011; Dodson et al. 2014; Hoffman et al. 2014; Meeker et al. 2013 b; Van den Eede et al. 2015). Die Kombination von APCI und ESI, die im negativen Ionisierungsmodus bei gleichzeitiger Verwendung des *Multiple Reaction Monitoring*arbeiten, ermöglichte mit dieser Methode eine selektive, empfindliche und robuste Quantifizierung von BDCPP in Urin.

Die Zuverlässigkeitskriterien der Methode können mit einer Präzision in der Serie von 3,1–6,5 % und einer Präzision von Tag zu Tag von 5,5–8,7 % als ausgezeichnet bezeichnet werden. Die Richtigkeit der Methode wurde durch hohe Wiederfindungen sowohl im gepoolten Urin als auch in Einzelurinen nach Aufstockung nachgewiesen. Außerdem ist die Selektivität der LC‑MS/MS-Methode sehr hoch. Signifikante Störpeaks wurden bei den für die Quantifizierung gewählten Massenübergängen nicht beobachtet.

Somit ermöglicht die Methode die empfindliche und valide Quantifizierung von BDCPP im Urin. Die Bestimmungs­grenze von 0,2 μg BDCPP pro Liter Urin ist für den Einsatz in der Arbeitsmedizin ausreichend und reicht teilweise für die Quantifizierung von Hintergrundgehalten in der Allgemeinbevölkerung aus. Um diese niedrige Bestimmungsgrenze zu erreichen, muss eine Multimode-Quelle eingesetzt werden, die eine gleichzeitige APCI und ESI ermöglicht.

**Verwendete Messgeräte **LC‑MS/MS‑System, verbunden mit einem Triple-Quadrupol‑Massenspektrometer mit einer Multimode-Quelle (Agilent Serie 1200 und Agilent 6460, Agilent Technologies, Inc., Santa Clara, CA, USA); Umkehr­phasensäule, Kinetex^®^core-shell silica (2,6 μm Biphenyl 100 Å, 100 × 2,1 mm) (Nr. 00D‑4622‑AN, Phenomenex Inc., Torrance, CA, USA) mit einer SecurityGuard-Vorsäule (Nr. AJ0‑9209, Phenomenex Inc., Torrance, CA, USA)

## Anmerkungen

Die ursprünglich entwickelte Methode (Krystek et al. 2019) war Teil eines auf 10 Jahre angelegten Projekts zur Weiterentwicklung des Human-Biomonitorings in Deutschland, das auf einer 2010 vereinbarten Kooperation zwischen dem Bundesministerium für Umwelt, Naturschutz und Reaktorsicherheit (BMU) und dem Verband der chemischen Industrie e. V. (VCI) beruhte.
